# Supplementary material for: Initial Evidence of Variation by Ethnicity in the Relationship between Vitamin C Status and Mental States in Young Adults
Source: Nutrients. 2021 Feb 27;13(3):792. doi: 10.3390/nu13030792 (PMC7997165; doi:10.3390/nu13030792)
Supplement: Supplementary file 1 [file nutrients-13-00792-s001.pdf]

Supplementary Material

Initial Evidence of Variation by Ethnicity in the Relationship Between Vitamin C Status and Mental States in Young Adults

Benjamin D. Fletcher[1], Shay-Ruby Wickham[1], Jayde A. M. Flett[2], Juliet M. Pullar[3], Margreet C. M. Vissers[3], & Tamlin S. Conner[1]

1. Department of Psychology, University of Otago, Dunedin, 9016, New Zealand.
2. Independent researcher, Wellington, New Zealand.
3. Centre for Free Radical Research, Department of Pathology and Biomedical Science, University of Otago, Christchurch, 8011, New Zealand.

CONTENTS

Supplementary Table 1. Number of Participants with Deficient (<11 μmol/L), Marginal (11-23 μmol/L), Inadequate (23-50 μmol/L), Adequate (50-70 μmol/L), or Saturated (70+ μmol/L) Vitamin C Levels for the Total Sample and Each Ethnicity and Gender Subgroup.

Supplementary Table 2. Number of Participants and Mean Vitamin C levels (μmol/L) for the Total Sample and Each Ethnicity by Gender Subgroup.

Supplementary Table 3. Regression Models Predicting Total Mood Disturbance, Vitality and Perceived Stress from the Demographic Covariates and Vitamin C Levels, using Europeans as the Reference Group, with Moderators.

Supplementary Table 4. Regression Models Predicting the Profile of Mood States (POMS) Subscales (tension, depression, anger, fatigue, confusion, and vigor) from the Demographic Covariates and Vitamin C Levels, using Europeans as the Reference Group, with Moderators.

Supplementary Table 5. Regression Models Predicting Total Mood Disturbance, Vitality and Perceived Stress from the Demographic Covariates and Vitamin C Levels, using Māori/Pasifika as the Reference Group, with Moderators.

Supplementary Table 6. Regression Models Predicting the Profile of Mood States (POMS) Subscales (tension, depression, anger, fatigue, confusion, and vigor) from the Demographic Covariates and Vitamin C Levels, using Māori/Pasifika as the Reference Group, with Moderators.

Supplementary Table 7. Regression Models Predicting Total Mood Disturbance, Vitality and Perceived Stress from the Demographic Covariates and Vitamin C Levels, using Southeast/Other Asians as the Reference Group, with Moderators.

Supplementary Table 8. Regression Models Predicting the Profile of Mood States (POMS) Subscales (tension, depression, anger, fatigue, confusion, and vigor) from the Demographic Covariates and Vitamin C Levels, using Southeast/Other Asians as the Reference Group, with Moderators.

Supplementary Table 1. *Number of Participants with Deficient (<11 μmol/L), Marginal (11-23 μmol/L), Inadequate (23-50 μmol/L), Adequate (50-70 μmol/L), or Saturated (70+ μmol/L) Vitamin C Levels for the Total Sample and Each Ethnicity and Gender Subgroup*

	Overall<br>*n*	Vitamin C<br>Mean (SD)	Deficient<br>n (%)	Marginal<br>n (%)	Inadequate<br>n (%)	Adequate<br>n (%)	Saturated<br>n (%)
Total Sample	419	54.90 (20.19)	4 (1.0%)	19 (4.5%)	140 (33.4%)	168 (40.1%)	88 (21.0%)
European	206	59.35 (19.35)	0 (0.0%)	6 (2.9%)	63 (30.6%)	80 (38.8%)	57 (27.7%)
East Asian	68	51.23 (19.16)	1 (1.5%)	3 (4.4%)	26 (38.2%)	29 (42.6%)	9 (13.2%)
Southeast/Other Asian	61	41.83 (18.62)	3 (4.9%)	5 (8.2%)	30 (49.2%)	19 (31.1%)	4 (6.6%)
Maori and Pasifika	38	60.96 (16.80)	0 (0.0%)	1 (2.6%)	7 (18.4%)	20 (52.6%)	10 (26.3%)
Other	46	52.76 (20.77)	0 (0.0%)	4 (8.7%)	14 (30.4%)	20 (43.5%)	8 (17.4%)
Gender							
Male	135	52.55 (18.46)	1 (0.7%)	8 (5.9%)	46 (34.1%)	60 (44.4%)	20 (14.8%)
Female	284	56.02 (20.90)	3 (1.1%)	11 (3.9%)	94 (33.1%)	108 (38.0%)	68 (23.9%)

Note. Percentage of participants displayed is out of the total number of participants per subgroup. SD = Standard Deviation

Supplementary Table 2. *Number of Participants and Mean Vitamin C levels (μmol/L) for the Total Sample and Each Ethnicity by Gender Subgroup.*

	Overall	Vitamin C in μmol/L			
		Male		Female	
	n	*n*	Mean (SD)	*n*	Mean (SD)
Total Sample	419	135	52.55 (18.46)	284	56.02 (20.89)
European	206	80	55.26 (18.57)	126	61.94 (19.72)
East Asian	68	17	46.83 (19.40)	51	52.69 (19.05)
Southeast/Other Asian	61	9	35.85 (12.29)	52	42.86 (19.41)
Maori and Pasifika	38	10	57.35 (8.50)	28	62.25 (18.87)
Other	46	19	51.63 (19.20)	27	53.55 (22.14)

Note. SD = Standard Deviation

Supplementary Table 3. *Regression Models Predicting Total Mood Disturbance, Vitality and Perceived Stress from the Demographic Covariates and Vitamin C Levels, using Europeans as the Reference Group, with Moderators*

	**Total Mood Disturbance**	**Vitality**	**Perceived stress**
	**b (SE)**	**b (SE)**	**b (SE)**
**Model 1- Unadjusted**			
Constant	9.876 (0.705)***	60.935 (0.738)***	5.305 (0.101)***
Vitamin C	0.002 (0.035)	0.013 (0.037)	-0.006 (0.005)
Overall R^2	0.000	0.000	0.004
**Model 2 - Adjusted**			
Constant	7.742 (1.341)***	62.258 (1.41)***	4.832 (0.185)***
Vitamin C	0.008 (0.036)	0.048 (0.038)	-0.003 (0.005)
SES	-2.585 (0.580)***	1.981 (0.610)**	-0.485 (0.080)***
Gender	-0.705 (1.53)	-1.089 (1.608)	-0.108 (0.211)
Age	-0.210 (0.221)	0.157 (0.232)	-0.026 (0.03)
Ethnicity D1 - East Asian	5.612 (2.052)**	-1.256 (2.157)	1.613 (0.284)***
Ethnicity D2 - Southeast Asian	3.183 (2.267)	2.318 (2.384)	0.857 (0.313)**
Ethnicity D3 - Māori/Pasifika	4.666 (2.487)†	-7.959 (2.615)**	0.720 (0.344)*
Ethnicity D4 - Other	7.420 (2.375)**	0.030 (2.497)	0.861 (0.328)**
R^2 Change	0.071***	0.062***	0.131***
F Change *(df)*	4.491 (7,410)	3.887 (7,410)	8.828 (7,410)
**Model 3- Adjusted + Moderators**			
Constant	7.756 (1.358)***	62.288 (1.411)***	4.787 (0.186)***
Vitamin C	0.008 (0.073)	0.121 (0.076)	-0.004 (0.010)
SES	-2.597 (0.584)***	1.941 (0.606)**	-0.482 (0.080)***
Gender	-0.742 (1.536)	-1.185 (1.596)	-0.109 (0.211)
Age	-0.225 (0.222)	0.180 (0.231)	-0.030 (0.031)
Ethnicity D1 - East Asian	5.715 (2.074)**	-1.277 (2.155)	1.632 (0.285)***
Ethnicity D2 - Southeast Asian	5.062 (2.538)*	-1.426 (2.637)	1.130 (0.348)**
Ethnicity D3 - Māori/Pasifika	5.497 (2.638)*	-9.511 (2.741)**	0.975 (0.362)**
Ethnicity D4 - Other	7.242 (2.384)**	0.301 (2.477)	0.863 (0.327)**
Vitamin C x Gender	-0.001 (0.079)	-0.067 (0.082)	0.012 (0.011)
Vitamin C x Ethnicity D1	0.022 (0.103)	-0.063 (0.107)	-0.011 (0.014)
Vitamin C x Ethnicity D2	0.141 (0.111)	-0.309 (0.116)**	0.009 (0.015)
Vitamin C x Ethnicity D3	-0.138 (0.148)	0.252 (0.154)	-0.044 (0.020)*
Vitamin C x Ethnicity D4	-0.100 (0.113)	0.080 (0.118)	-0.027 (0.016)†
R^2 Change	0.009	0.032*	0.020†
F Change *(df)*	0.826 (5,405)	2.821 (5,405)	1.879 (5,405)
Overall R^2	0.081	0.094	0.154

Note. b (SE) = unstandardized coefficient (Standard Error), SES = socioeconomic status, D = dummy code, † $p < .10$ * $p < .05$; ** $p < .01$; *** $p < .001$.

Supplementary Table 4. *Regression Models Predicting the Profile of Mood States (POMS) Subscales (tension, depression, anger, fatigue, confusion, and vigor) from the Demographic Covariates and Vitamin C Levels, using Europeans as the Reference Group, with Moderators*

	Tension b (SE)	Depression b (SE)	Anger b (SE)	Fatigue b (SE)	Confusion b (SE)	Vigor b (SE)
**Model 1 - Unadjusted**						
Constant	4.792 (0.170)***	2.286 (0.128)***	2.644 (0.131)***	6.399 (0.186)***	4.721 (0.156)***	10.967 (0.178)***
Vitamin C	0.004 (0.008)	-0.006 (0.006)	-0.012 (0.007)†	0.013 (0.009)	0.001 (0.008)	-0.001 (0.009)
Overall R2	0.001	0.002	0.008†	0.005	0.000	0.000
**Model 2 - Adjusted**						
Constant	4.386 (0.327)***	1.829 (0.240)***	2.385 (0.252)***	6.241 (0.360)***	4.539 (0.296)***	11.639 (0.340)***
Vitamin C	0.009 (0.009)	0.000 (0.006)	-0.007 (0.007)	0.009 (0.010)	0.003 (0.008)	0.007 (0.009)
SES	-0.527 (0.141)***	-0.488 (0.104)***	-0.266 (0.109)*	-0.447 (0.156)**	-0.601 (0.128)***	0.255 (0.147)†
Gender	-0.354 (0.372)	-0.247 (0.274)	-0.401 (0.288)	0.032 (0.411)	-0.464 (0.338)	-0.729 (0.388)†
Age	-0.071 (0.054)	-0.001 (0.040)	0.015 (0.042)	-0.032 (0.059)	-0.083 (0.049)†	0.038 (0.056)
Ethnicity D1 - East Asian	1.146 (0.500)*	1.284 (0.368)**	0.961 (0.386)*	-0.137 (0.551)	0.899 (0.453)*	-1.458 (0.520)**
Ethnicity D2 - Southeast Asian	1.459 (0.552)**	1.112 (0.407)**	0.914 (0.427)*	-0.007 (0.609)	0.913 (0.500)†	1.208 (0.574)*
Ethnicity D3 - Māori/Pasifika	0.570 (0.606)	0.389 (0.446)	1.141 (0.468)*	1.248 (0.668)†	0.441 (0.549)	-0.876 (0.630)
Ethnicity D4 - Other	1.786 (0.578)**	1.996 (0.426)***	1.261 (0.447)**	0.420 (0.638)	1.613 (0.524)**	-0.343 (0.602)
R2 Change	0.057**	0.098***	0.048**	0.034*	0.072***	0.062***
F Change (df)	3.554 (7,410)	6.396 (7,410)	2.965 (7,410)	2.097 (7,410)	4.537 (7,410)	3.861 (7,410)
**Model 3 - Adjusted + Moderators**						
Constant	4.370 (0.331)***	1.782 (0.244)***	2.386 (0.257)***	6.252 (0.365)***	4.573 (0.299)***	11.607 (0.343)***
Vitamin C	0.026 (0.018)	-0.002 (0.013)	-0.012 (0.014)	-0.008 (0.020)	0.011 (0.016)	0.006 (0.018)
SES	-0.535 (0.142)***	-0.483 (0.105)***	-0.263 (0.110)*	-0.438 (0.157)**	-0.612 (0.128)***	0.265 (0.147)†
Gender	-0.372 (0.374)	-0.247 (0.276)	-0.398 (0.290)	0.061 (0.412)	-0.486 (0.338)	-0.699 (0.388)†
Age	-0.068 (0.054)	-0.006 (0.040)	0.012 (0.042)	-0.034 (0.060)	-0.084 (0.049)†	0.046 (0.056)
Ethnicity D1 - East Asian	1.108 (0.505)*	1.294 (0.373)**	0.999 (0.392)*	-0.184 (0.557)	0.914 (0.456)*	-1.583 (0.524)**
Ethnicity D2 - Southeast Asian	1.742 (0.618)**	1.161 (0.456)*	0.917 (0.480)†	0.616 (0.681)	1.474 (0.558)**	0.847 (0.641)
Ethnicity D3 - Māori/Pasifika	0.901 (0.642)	0.472 (0.474)	1.129 (0.499)*	1.376 (0.708)†	0.639 (0.580)	-0.979 (0.666)
Ethnicity D4 - Other	1.808 (0.580)**	2.013 (0.429)***	1.243 (0.451)**	0.378 (0.640)	1.551 (0.524)**	-0.249 (0.602)
Vitamin C x Gender	-0.010 (0.019)	0.013 (0.014)	0.005 (0.015)	0.011 (0.021)	-0.013 (0.017)	0.006 (0.020)
Vitamin C x Ethnicity D1	-0.031 (0.025)	-0.013 (0.019)	0.014 (0.019)	0.009 (0.028)	0.006 (0.023)	-0.036 (0.026)
Vitamin C x Ethnicity D2	0.011 (0.027)	-0.008 (0.020)	0.002 (0.021)	0.059 (0.030)†	0.047 (0.024)†	-0.031 (0.028)
Vitamin C x Ethnicity D3	-0.057 (0.036)	-0.016 (0.027)	0.002 (0.028)	-0.019 (0.040)	-0.032 (0.033)	0.016 (0.037)
Vitamin C x Ethnicity D4	-0.013 (0.028)	-0.020 (0.020)	-0.006 (0.021)	0.006 (0.030)	-0.024 (0.025)	0.044 (0.029)
R2 Change	0.011	0.005	0.002	0.012	0.016	0.016
F Change (df)	0.953 (5,405)	0.412 (5,405)	0.175 (5,405)	1.031 (5,405)	1.461 (5,405)	1.427 (5,405)
Overall R2	0.069	0.105	0.057	0.052	0.088	0.078

Note. b (SE) = unstandardized coefficient (Standard Error), SES = socioeconomic status, D = dummy code, † $p < .10$ * $p < .05$; ** $p < .01$; *** $p < .001$.

Supplementary Table 5. *Regression Models Predicting Total Mood Disturbance, Vitality and Perceived Stress from the Demographic Covariates and Vitamin C Levels, using Māori/Pasifika as the Reference Group, with Moderators*

	Total Mood Disturbance<br>b (SE)	Vitality<br>b (SE)	Perceived stress<br>b (SE)
Model 1 - Adjusted			
Constant	12.408 (2.535)***	54.299 (2.665)***	5.553 (0.350)***
Vitamin C	0.008 (0.036)	0.048 (0.038)	-0.003 (0.005)
SES	-2.585 (0.580)***	1.981 (0.610)**	-0.485 (0.80)***
Gender	-0.705 (1.530)	-1.089 (1.608)	-0.108 (0.211)
Age	-0.210 (0.221)	0.157 (0.232)	-0.026 (0.030)
Ethnicity D1 - European	-4.666 (2.487)†	7.959 (2.615)**	-0.720 (0.344)*
Ethnicity D2 - East Asian	0.946 (2.919)	6.703 (3.069)*	0.893 (0.403)*
Ethnicity D3 - Southeast Asian	-1.483 (3.074)	10.278 (3.232)**	0.137 (0.425)
Ethnicity D4 - Other	2.754 (3.173)	7.990 (3.336)*	0.141 (0.438)
R^2 Change	0.071***	0.063**	0.134***
F Change (*df*)	3.93 (8,410)	3.419 (8,410)	7.938 (8,410)
Model 2 - Adjusted + Moderators			
Constant	13.253 (2.664)***	52.777 (2.767)***	5.762 (0.366)***
Vitamin C	-0.130 (0.157)	0.373 (0.163)*	-0.048 (0.022)*
SES	-2.597 (0.584)***	1.941 (0.606)**	-0.482 (0.080)***
Gender	-0.742 (1.536)	-1.185 (1.596)	-0.109 (0.211)
Age	-0.225 (0.222)	0.180 (0.231)	-0.030 (0.031)
Ethnicity D1 - European	-5.497 (2.638)*	9.511 (2.741)**	-0.975 (0.362)**
Ethnicity D2 - East Asian	0.218 (3.044)	8.233 (3.162)*	0.657 (0.418)
Ethnicity D3 - Southeast Asian	-0.435 (3.374)	8.084 (3.505)*	0.155 (0.463)
Ethnicity D4 - Other	1.744 (3.280)	9.812 (3.408)**	-0.112 (0.450)
Vitamin C x Gender	-0.001 (0.079)	-0.067 (0.082)	0.012 (0.011)
Vitamin C x Ethnicity D1	0.138 (0.148)	-0.252 (0.154)	0.044 (0.020)*
Vitamin C x Ethnicity D2	0.160 (0.165)	-0.315 (0.171)†	0.033 (0.023)
Vitamin C x Ethnicity D3	0.279 (0.169)†	-0.560 (0.175)**	0.053 (0.023)*
Vitamin C x Ethnicity D4	0.038 (0.173)	-0.171 (0.179)	0.017 (0.024)
R^2 Change	0.009	0.032*	0.020†
F Change (*df*)	0.826 (5,405)	2.821 (5,405)	1.879 (5,405)
Overall R^2	0.081	0.094	0.154

Note. b (SE) = unstandardized coefficient (Standard Error), SES = socioeconomic status, D = dummy code, † $p < .10$ * $p < .05$; ** $p < .01$; *** $p < .001$.

Supplementary Table 6. *Regression Models Predicting the Profile of Mood States (POMS) Subscales (tension, depression, anger, fatigue, confusion, and vigor) from the Demographic Covariates and Vitamin C Levels, using Māori/Pasifika as the Reference Group, with Moderators*

	Tension<br>b (SE)	Depression<br>b (SE)	Anger<br>b (SE)	Fatigue<br>b (SE)	Confusion<br>b (SE)	Vigor<br>b (SE)
**Model 1 - Adjusted**						
Constant	4.956 (0.617)***	2.218 (0.455)***	3.526 (0.477)***	7.489 (0.681)***	4.98 (0.559)***	10.763 (0.642)***
Vitamin C	0.009 (0.009)	0.000 (0.006)	-0.007 (0.007)	0.009 (0.010)	0.003 (0.008)	0.007 (0.009)
SES	-0.527 (0.141)***	-0.488 (0.104)***	-0.266 (0.109)*	-0.447 (0.156)**	-0.601 (0.128)***	0.255 (0.147)†
Gender	-0.354 (0.372)	-0.247 (0.274)	-0.401 (0.288)	0.032 (0.411)	-0.464 (0.338)	-0.729 (0.388)†
Age	-0.071 (0.054)	-0.001 (0.040)	0.015 (0.042)	-0.032 (0.059)	-0.083 (0.049)†	0.038 (0.056)
Ethnicity D1 - European	-0.570 (0.606)	-0.389 (0.446)	-1.141 (0.468)*	-1.248 (0.668)†	-0.441 (0.549)	0.876 (0.630)
Ethnicity D2 - East Asian	0.576 (0.711)	0.895 (0.524)†	-0.180 (0.550)	-1.385 (0.785)†	0.458 (0.644)	-0.582 (0.740)
Ethnicity D3 - Southeast Asian	0.889 (0.749)	0.722 (0.551)	-0.227 (0.579)	-1.255 (0.826)	0.471 (0.678)	2.084 (0.779)**
Ethnicity D4 - Other	1.216 (0.773)	1.607 (0.569)**	0.120 (0.597)	-0.828 (0.853)	1.172 (0.700)†	0.533 (0.804)
R2 Change	0.058**	0.100***	0.055**	0.039*	0.072***	0.062**
F Change (df)	3.138 (8,410)	5.702 (8,410)	3.004 (8,410)	2.105 (8,410)	3.973 (8,410)	3.379 (8,410)
**Model 2 – Adjusted + Moderators**						
Constant	5.271 (0.648)***	2.254 (0.479)***	3.515 (0.503)***	7.628 (0.715)***	5.212 (0.586)***	10.628 (0.673)***
Vitamin C	-0.031 (0.038)	-0.018 (0.028)	-0.010 (0.030)	-0.027 (0.042)	-0.021 (0.034)	0.022 (0.040)
SES	-0.535 (0.142)***	-0.483 (0.105)***	-0.263 (0.110)*	-0.438 (0.157)**	-0.612 (0.128)***	0.265 (0.147)†
Gender	-0.372 (0.374)	-0.247 (0.276)	-0.398 (0.290)	0.061 (0.412)	-0.486 (0.338)	-0.699 (0.388)†
Age	-0.068 (0.054)	-0.006 (0.040)	0.012 (0.042)	-0.034 (0.060)	-0.084 (0.049)†	0.046 (0.056)
Ethnicity D1 - European	-0.901 (0.642)	-0.472 (0.474)	-1.129 (0.499)*	-1.376 (0.708)†	-0.639 (0.580)	0.979 (0.666)
Ethnicity D2 - East Asian	0.207 (0.741)	0.821 (0.547)	-0.130 (0.575)	-1.560 (0.817)†	0.275 (0.669)	-0.604 (0.768)
Ethnicity D3 - Southeast Asian	0.840 (0.821)	0.688 (0.607)	-0.213 (0.638)	-0.761 (0.906)	0.835 (0.742)	1.826 (0.852)*
Ethnicity D4 - Other	0.906 (0.798)	1.540 (0.590)**	0.114 (0.620)	-0.998 (0.881)	0.912 (0.721)	0.730 (0.828)
Vitamin C x Gender	-0.010 (0.019)	0.013 (0.014)	0.005 (0.015)	0.011 (0.021)	-0.013 (0.017)	0.006 (0.020)
Vitamin C x Ethnicity D1	0.057 (0.036)	0.016 (0.027)	-0.002 (0.028)	0.019 (0.040)	0.032 (0.033)	-0.016 (0.037)
Vitamin C x Ethnicity D2	0.026 (0.040)	0.002 (0.030)	0.012 (0.031)	0.028 (0.044)	0.038 (0.036)	-0.053 (0.042)
Vitamin C x Ethnicity D3	0.068 (0.041)†	0.008 (0.030)	0.000 (0.032)	0.078 (0.045)†	0.079 (0.037)*	-0.047 (0.043)
Vitamin C x Ethnicity D4	0.044 (0.042)	-0.004 (0.031)	-0.008 (0.033)	0.026 (0.046)	0.008 (0.038)	0.028 (0.044)
R2 Change	0.011	0.005	0.002	0.012	0.016	0.016
F Change (df)	0.953 (5,405)	0.412 (5,405)	0.175 (5,405)	1.031 (5,405)	1.461 (5,405)	1.427 (5,405)
Overall R2	0.069	0.105	0.057	0.052	0.088	0.078

Note. b (SE) = unstandardized coefficient (Standard Error), SES = socioeconomic status, D = dummy code, † $p < .10$ * $p < .05$; ** $p < .01$; *** $p < .001$.

Supplementary Table 7. *Regression Models Predicting Total Mood Disturbance, Vitality and Perceived Stress from the Demographic Covariates and Vitamin C Levels, using Southeast/Other Asians as the Reference Group, with Moderators*

	Total Mood Disturbance	Vitality	Perceived stress
	b (SE)	b (SE)	b (SE)
Model 1 - Adjusted			
Constant	10.925 (2.363)***	64.576 (2.484)***	5.689 (0.327)***
Vitamin C	0.008 (0.036)	0.048 (0.038)	-0.003 (0.005)
SES	-2.585 (0.580)***	1.981 (0.610)**	-0.485 (0.080)***
Gender	-0.705 (1.530)	-1.089 (1.608)	-0.108 (0.211)
Age	-0.210 (0.221)	0.157 (0.232)	-0.026 (0.030)
Ethnicity D1 – European	-3.183 (2.267)	-2.318 (2.384)	-0.857 (0.313)**
Ethnicity D2 – East Asian	2.429 (2.527)	-3.575 (2.656)	0.756 (0.349)*
Ethnicity D3 – Māori/Pasifika	1.483 (3.074)	-10.278 (3.232)**	-0.137 (0.425)
Ethnicity D4 – Other	4.237 (2.805)	-2.288 (2.950)	0.004 (0.388)
R2 Change	0.071***	0.063**	0.134***
F Change (df)	3.930 (8,410)	3.419 (8,410)	7.938 (8,410)
Model 2 Adjusted + Moderators			
Constant	12.818 (2.648)***	60.862 (2.751)***	5.917 (0.364)***
Vitamin C	0.149 (0.121)	-0.188 (0.125)	0.005 (0.017)
SES	-2.597 (0.584)***	1.941 (0.606)**	-0.482 (0.080)***
Gender	-0.742 (1.536)	-1.185 (1.596)	-0.109 (0.211)
Age	-0.225 (0.222)	0.180 (0.231)	-0.030 (0.031)
Ethnicity D1 – European	-5.062 (2.538)*	1.426 (2.637)	-1.130 (0.348)**
Ethnicity D2 – East Asian	0.653 (2.830)	0.149 (2.940)	0.502 (0.389)
Ethnicity D3 – Māori/Pasifika	0.435 (3.374)	-8.084 (3.505)*	-0.155 (0.463)
Ethnicity D4 – Other	2.180 (3.067)	1.727 (3.186)	-0.267 (0.421)
Vitamin C x Gender	-0.001 (0.079)	-0.067 (0.082)	0.012 (0.011)
Vitamin C x Ethnicity D1	-0.141 (0.111)	0.309 (0.116)**	-0.009 (0.015)
Vitamin C x Ethnicity D2	-0.119 (0.133)	0.246 (0.138)†	-0.020 (0.018)
Vitamin C x Ethnicity D3	-0.279 (0.169)†	0.560 (0.175)**	-0.053 (0.023)*
Vitamin C x Ethnicity D4	-0.241 (0.142)†	0.389 (0.147)**	-0.036 (0.019)†
R2 Change	0.009	0.032*	0.020†
F Change (df)	0.826 (5,405)	2.821 (5,405)	1.879 (5,405)
Overall R2	0.081	0.094	0.154

Note. b (SE) = unstandardized coefficient (Standard Error), SES = socioeconomic status, D = dummy code, † $p < .10$ * $p < .05$; ** $p < .01$; *** $p < .001$.

Supplementary Table 8. *Regression Models Predicting the Profile of Mood States (POMS) Subscales (tension, depression, anger, fatigue, confusion, and vigor) from the Demographic Covariates and Vitamin C Levels, using Southeast/Other Asians as the Reference Group, with Moderators*

	Tension<br>b (SE)	Depression<br>b (SE)	Anger<br>b (SE)	Fatigue<br>b (SE)	Confusion<br>b (SE)	Vigor<br>b (SE)
**Model 1 - Adjusted**						
Constant	5.845 (0.575)***	2.941 (0.424)***	3.299 (0.445)***	6.234 (0.635)***	5.452 (0.521)***	12.846 (0.599)***
Vitamin C	0.009 (0.009)	0.000 (0.006)	-0.007 (0.007)	0.009 (0.010)	0.003 (0.008)	0.007 (0.009)
SES	-0.527 (0.141)***	-0.488 (0.104)***	-0.266 (0.109)*	-0.447 (0.156)**	-0.601 (0.128)***	0.255 (0.147)†
Gender	-0.354 (0.372)	-0.247 (0.274)	-0.401 (0.288)	0.032 (0.411)	-0.464 (0.338)	-0.729 (0.388)†
Age	-0.071 (0.054)	-0.001 (0.040)	0.015 (0.042)	-0.032 (0.059)	-0.083 (0.049)†	0.038 (0.056)
Ethnicity D1 - European	-1.459 (0.552)**	-1.112 (0.407)**	-0.914 (0.427)*	0.007 (0.609)	-0.913 (0.500)†	-1.208 (0.574)*
Ethnicity D2 - East Asian	-0.313 (0.615)	0.172 (0.453)	0.047 (0.476)	-0.130 (0.679)	-0.013 (0.558)	-2.666 (0.640)***
Ethnicity D3 - Māori/Pasifika	-0.889 (0.749)	-0.722 (0.551)	0.227 (0.579)	1.255 (0.826)	-0.471 (0.678)	-2.084 (0.779)**
Ethnicity D4 - Other	0.327 (0.683)	0.885 (0.503)†	0.347 (0.528)	0.427 (0.754)	0.701 (0.619)	-1.551 (0.711)*
R2 Change	0.058**	0.100***	0.055**	0.039*	0.072***	0.062**
F Change (df)	3.138 (8,410)	5.702 (8,410)	3.004 (8,410)	2.105 (8,410)	3.973 (8,410)	3.379 (8,410)
**Model 2 – Adjusted + Moderators**						
Constant	6.112 (0.644)***	2.943 (0.476)***	3.302 (0.500)***	6.868 (0.711)***	6.047 (0.582)***	12.454 (0.668)***
Vitamin C	0.037 (0.029)	-0.010 (0.022)	-0.011 (0.023)	0.051 (0.032)	0.057 (0.027)*	-0.025 (0.030)
SES	-0.535 (0.142)***	-0.483 (0.105)***	-0.263 (0.110)*	-0.438 (0.157)**	-0.612 (0.128)***	0.265 (0.147)†
Gender	-0.372 (0.374)	-0.247 (0.276)	-0.398 (0.290)	0.061 (0.412)	-0.486 (0.338)	-0.699 (0.388)†
Age	-0.068 (0.054)	-0.006 (0.040)	0.012 (0.042)	-0.034 (0.060)	-0.084 (0.049)†	0.046 (0.056)
Ethnicity D1 - European	-1.742 (0.618)**	-1.161 (0.456)*	-0.917 (0.480)†	-0.616 (0.681)	-1.474 (0.558)**	-0.847 (0.641)
Ethnicity D2 - East Asian	-0.633 (0.689)	0.133 (0.509)	0.083 (0.535)	-0.799 (0.760)	-0.560 (0.622)	-2.430 (0.714)**
Ethnicity D3 - Māori/Pasifika	-0.840 (0.821)	-0.688 (0.607)	0.213 (0.638)	0.761 (0.906)	-0.835 (0.742)	-1.826 (0.852)*
Ethnicity D4 - Other	0.066 (0.746)	0.852 (0.551)	0.326 (0.580)	-0.238 (0.823)	0.077 (0.674)	-1.096 (0.774)
Vitamin C x Gender	-0.010 (0.019)	0.013 (0.014)	0.005 (0.015)	0.011 (0.021)	-0.013 (0.017)	0.006 (0.020)
Vitamin C x Ethnicity D1	-0.011 (0.027)	0.008 (0.020)	-0.002 (0.021)	-0.059 (0.030)†	-0.047 (0.024)†	0.031 (0.028)
Vitamin C x Ethnicity D2	-0.042 (0.032)	-0.005 (0.024)	0.013 (0.025)	-0.049 (0.036)	-0.041 (0.029)	-0.006 (0.034)
Vitamin C x Ethnicity D3	-0.068 (0.041)†	-0.008 (0.030)	0.000 (0.032)	-0.078 (0.045)†	-0.079 (0.037)*	0.047 (0.043)
Vitamin C x Ethnicity D4	-0.024 (0.035)	-0.012 (0.025)	-0.007 (0.027)	-0.052 (0.038)	-0.071 (0.031)*	0.075 (0.036)*
R2 Change	0.011	0.005	0.002	0.012	0.016	0.016
F Change (df)	0.953 (5,405)	0.412 (5,405)	0.175 (5,405)	1.031 (5,405)	1.461 (5,405)	1.427 (5,405)
Overall R2	0.069	0.105	0.057	0.052	0.088	0.078

Note. b (SE) = unstandardized coefficient (Standard Error), SES = socioeconomic status, D = dummy code, † *p* < .10 * *p* < .05; ** *p* < .01; *** *p* < .001.
